# The Treatment of an Impending Calamity

**Published:** 1908-08-29

**Authors:** 


### Anæsthetics.

### THE TREATMENT OF AN IMPENDING CALAMITY.

The ideal treatment of grave difficulties during the exhibition of anæsthetic drugs is naturally the prophylactic. Both teachers and text-books rightly press home this point with the greatest possible emphasis, for it is far more important than the curative treatment of that which has already occurred. But however practised and careful the anæsthetist may be, it is certain that at intervals, which usually become rarer as experience increases, he will be confronted with the necessity for taking active measures to avert disaster. There are two broad principles by which the anæsthetist must rule his conduct whenever the condition of his patient gives rise to anxiety. He must have a clear conception of what measures he proposes to take, of the order in which he proposes to take them, and of the special needs of the particular case; but, while these must ever be fixed firmly in his mind and no emotion allowed to confuse them, it is almost equally essential that his judgment shall be calm and accurate as to the necessity for employing them or otherwise. The anæsthetist who worries surgeons by requests for artificial respiration when there is no need for it will not be regarded with favour; though he is, needless to say, far less culpable than is one who neglects the early signs of overdose or other complication.

It is impossible to deal in a brief space with all the varying sources of difficulty which an anæsthetist may have to contend with. Let us assume, therefore, that the maintenance of an adequate and patent airway is secured, that the operative procedures have in no way interfered with the administration, that the respiratory centre and the other basal ganglia are the seat of no gross disease or disability; in short, let us consider the case of a patient undergoing an abdominal section or an amputation to whom danger has arisen from the action of the anæsthetic. Almost invariably in such a case the exhibited drug is chloroform, pure or in a mixture, and for practical purposes the result may be regarded as due to an overdose, actual or "relative," of that substance. By relative overdose is understood such a condition of deficient respiratory interchange as renders dangerous in the patient's circulation a quantity of chloroform which would not otherwise imperil his vital functions.*

Whatever the deductions of chloroform Commissions at various times, it is probably commoner, when anæsthetics are being administered to human beings, for respiration to fail before circulation, apart from those mysterious cases of sudden death during the induction stage, when chloroform is administered to excess.

The first sign of such excess is nearly always a gradual failure of respiration, which becomes shallower and shallower, irregular, and finally imperceptible. The colour of the patient during this process is of but little value in determining whether the onset of shallow respiration is due to too much chloroform or to the imminent return of semi-consciousness from too little. In the latter case the patient may be pale from slight shock induced by the operative proceedings: in the former a similar pallor may be at first observed, or the colour may for a short time be fairly well maintained; if a "relative" overdose only is the cause of the symptoms, there may be marked cyanosis from the beginning, and in any case this sign supervenes ultimately in cases of real overdose, causing respiratory failure, which are not promptly treated. The pulse is of much less value as a diagnostic sign. It is sometimes quite strong and regular when respiration has already just ceased from overdose: at other times it fails as soon as, or almost as soon as, the breathing. Moreover, it is affected by many other things beside the anæsthetic. The eye is even less reliable: of course, an active lid reflex is hardly compatible with even relative overdose, but the state of the pupil is not much guide to the degree of toxæmia. In many subjects, as is well known, a widely dilated fixed pupil accompanies deep chloroform toxæmia, but this is very commonly not accompanied by any effect on respiration; and it is equally the case that a contracted pupil is no security against imminent respiratory failure.

The very first thing to do, after suspending the administration, is to take measures to restore the respiratory function. If the jaws are not already propped widely apart, a gag should be put in and opened nearly to its full extent. In mild cases of early failure of respiration brisk rubbing of the lips with a dry towel and pulling forward the tongue by inserting a forefinger down to its base and hooking the whole organ towards the chin by downward pressure may be all that is necessary: the latter manœuvre also ensures a clear airway. At the same time it is a useful plan to lower the head and raise the feet. In dealing with babies it is recommended to hold them up by the ankles. The next measure, if these are not speedily successful, is to apply rhythmic tongue traction: this necessitates the application of tongue forceps, admittedly barbarous weapons which should never be used except in pressing urgency, but efficient and useful in some of the cases under consideration. At the same time someone should be instructed to fetch and make ready the oxygen cylinder, if one is handy; and here it may be noted that no hospital theatre should be regarded as fully equipped unless provided with this apparatus, kept ready for immediate use. In private the habit of carrying an oxygen cylinder about is not likely to be generally adopted; but all nursing homes should be provided with this appliance, and in a hospital there is no excuse for its absence; we lay especial stress on this because in London, and doubtless elsewhere in these islands, there are hospital theatres quite unprovided with the means of administering oxygen.

If none of these measures quickly restores efficient respiration, the anæsthetist must announce to the surgeon that he intends at once to employ artificial respiration. The latter will cover with gauze or

* *Vide* "Practical Anæsthetics," by H. E. G. Boyle, Pp. 101 *et seq.*

other dressing the field of operation, and to him or his assistants should be allotted the duty of giving a hypodermic injection of strychnine (ten or twelve minims at least) or of ether (a drachm). The nurses must be busied in procuring very hot towels, which are to be applied to the præcordium; and in delivering oxygen, through a glass funnel preferably, to the immediate neighbourhood of the patient's mouth and nose. Someone must hold the gag in position and the tongue well forward with tongue forceps, while the anæsthetist applies himself to compression of the chest. The object of compressing the chest before taking any step to promote inspiration is to expel, in the first instance, as much of the chloroform vapour from the lungs as possible. If the patient is already supine, he had best remain so, and the surgeon may compress the abdomen while the anæsthetist does the like to the thorax. On releasing the pressure the ribs will expand a certain amount, and it is customary to bring the arms from a folded position on the chest to the extended attitude over the head in order to assist the inspiratory movement. The commonest mistake is to go through this cycle too frequently: probably ten or twelve times a minute is quite often enough, but in the agitation of the moment that rate is frequently much exceeded.

While artificial respiration is being thus conducted, it is worth while to have the limbs bandaged from below upwards if there is a spare assistant to whom this can be entrusted: this "auto-transfusion" is held to be of particular value in collapse from loss of blood and similar causes, and in such cases saline infusion through the median basilic vein is also valuable. When deep cyanosis complicates the symptoms, it has been recommended to allow several ounces of blood to escape from the selected vein before inserting the transfusion cannula; this obviously presupposes a strongly-acting heart.

It must not be forgotten that in rare cases the commencement of an operation under too light an anæsthesia may produce an effect on the respiratory or cardiac centres, or both, which results in cessation of their functions. Laryngeal spasm due to a similar reflex is perhaps less uncommon, and equally disastrous. The moral is that the anæsthetist, when confronted with such a case, should take the most energetic measures to meet these symptoms of shock, and auto-transfusion and strychnine injections assume a greater relative value than in dealing with simple overdose.

In these cases one would be inclined to refrain from using a nitrite of amyl capsule, because the blood-pressure is already so low that any vasodilator effect will merely add to the embarrassment of the heart. What is wanted is to supply the heart with the lacking stimulus to contraction, and that is blood or something as similar as possible, such as saline solution. The correct use of amyl nitrite is in cases of slight cyanosis with "relative" overdose: for then, if the heart has not yet failed, the respiratory centre may thus be supplied with an extra quantity of the insufficiently oxygenised blood to make up for its inferior quality; at the same time the degree of toxæmia due to contained chloroform is not increased, because probably the percentage in the blood is lethal merely in the absence of due aëration.

The comparative value of the various proceedings which have been enumerated is not easy to indicate, because they are used one after the other so quickly that a fortunate result may be due to any of them or to the combination. The practice of applying hot cloths to the præcordium is a well-established one, though what precise good it does is difficult to say: no direct heat can reach the heart, nor is it clear that any useful purpose would be served if it did; the effect, if any, must be reflex. No time should be wasted over this item if helping hands are few, though it should certainly not be neglected if plenty of assistance is available. The sheet-anchor in anæsthetic calamities is artificial respiration, and in all cases it must be kept up for half or three-quarters of an hour at least: this, with oxygen and the supply of warmth by hot bottles and blankets, is the most reliable resort in desperate cases, though it must be mentioned that in a few apparently desperate instances massage of the heart through the diaphragm and acupuncture of the same viscus through the chest have been known to succeed.

